# The estimated mediating roles of anemia-related variables in the association between kidney function and mortality: a National Health and Nutrition Examination Survey (NHANES) study

**DOI:** 10.1038/s41598-024-56877-7

**Published:** 2024-03-19

**Authors:** Yae Hyun Kim, Whanhee Lee, Kyun Young Kim, Yaerim Kim, Ara Ko, Boram Weon, Jeonghwan Lee, Wencheng Jin, Dong Ki Kim, Yon Su Kim, Chun Soo Lim, Jung Pyo Lee, Korean Association for the study of Renal Anemia, artificial Intelligence (KARAI), Sung Gyun Kim, Gang Jee Ko, Jung Tak Park, Tae Ik Chang, Sungjin Chung, Sang Ho Lee, Bum Soon Choi, Jin Seok Jeon, Sangheon Song, Dae Eun Choi, Dong‑Ryeol Ryu, Woo Kyung Jung

**Affiliations:** 1 Department of Internal Medicine, Seoul National University Hospital, Seoul, Korea; 2 School of Biomedical Convergence Engineering, Pusan National University College of Information and Biomedical Engineering, Pusan, Korea; 3 Department of Internal Medicine, Keimyung University School of Medicine, Taegu, Korea; 4 Department of Internal Medicine, Seoul National University Boramae Medical Center, 20 Boramae-ro 5-gil, Dongjak-gu, Seoul, 07061 Republic of Korea; 5 Department of Internal Medicine, Seoul National University College of Medicine, Seoul, Korea; 6 Hallym University College of Medicine, Anyang, Korea; 7 Korea University College of Medicine, Seoul, Korea; 8 Yonsei University College of Medicine, Seoul, Korea; 9 National Health Insurance Service Ilsan Hospital, Goyang, Korea; 10 College of Medicine, The Catholic University of Korea, Seoul, Korea; 11 College of Medicine, Kyung Hee University, Seoul, Korea; 12 Soon Chun Hyang University, Seoul, Korea; 13 Pusan National University College of Medicine, Pusan, Korea; 14 Chungnam National University College of Medicine, Taejon, Korea; 15 Division of Nephrology, Department of Internal Medicine, School of Medicine, Ehwa Womans University, Seoul, Republic of Korea; 16 Gachon University of Medicine and Science, Inchon, Korea

**Keywords:** Chronic kidney disease, Anemia, Mediation analysis, Mortality, Anaemia, Nephrology, Kidney, Kidney diseases

## Abstract

Anemia is a common complication of chronic kidney disease (CKD), impacting long-term outcomes such as mortality and morbidity. Analyzing NHANES data from 1999 through 2016 for adults aged ≥ 20 years, we assessed the mediating effects of anemia biomarkers (hemoglobin, hematocrit, red cell distribution width [RDW], and mean corpuscular hemoglobin concentration [MCHC]) on CKD-related outcomes by using hazard ratios from a biomarker-adjusted model. Of 44,099 participants, 7463 experienced all-cause death. Cox proportional hazard models revealed a higher all-cause mortality risk in the > 45 years and CKD groups than in the early CKD group. Hemoglobin, hematocrit and MCHC were inversely related to all-cause mortality; RDW was related to mortality. Single mediation analysis showed greater mediating effects of anemia indicators on CKD and mortality in the elderly (> 65 years) population than those in the general population. In the multimediation analysis, the combined mediating effect of anemia was higher in the CKD population than in the general population. This study showed a proportional increase in the mediating effect of anemia with CKD stage, suggesting potential therapeutic avenues. However, further exploration of other mediating factors on kidney outcomes is necessary.

## Introduction

Chronic kidney disease (CKD) is a common disease with a global estimated prevalence of 13.4% (11.7–15.1%), and the number of patients with end-stage kidney disease (ESKD) needing renal replacement therapy is estimated to be between 4.902 and 7.083 million^1^. The presence of CKD increases the risk of developing cardiovascular (CV) disease, hyperlipidemia, mineral and bone disorders, and anemia^2^. Among these conditions, anemia is one of the most common complications in CKD patients^2^. In addition, anemia affects the quality of life, cognitive function, physical performance, and cardiac function of patients and leads to deterioration of kidney function and mortality^3–5^. However, many studies have suggested that complete correction of anemia neither reduces the risk of cardiovascular and thrombosis risk^6–8^ nor improves outcomes in CKD patients with anemia^6–9^. It is known that there is a U-shaped association between the level of hemoglobin concentration and all-cause mortality^10–12^. Therefore, the current guidelines for CKD patients with anemia do not recommend normalization of hemoglobin levels^13,14^.

It is not easy to assess the effect of anemia on long-term CKD outcomes, such as mortality and morbidity, because kidney function affects outcomes directly and indirectly. Additionally, whether the relationship between CKD and clinical outcomes is mediated in part or fully by renal anemia is not known in detail. Hence, in this study, we used mediation analysis as a new statistical technique to explore the mediating effects of anemia on kidney outcomes.

**Figure 1:**
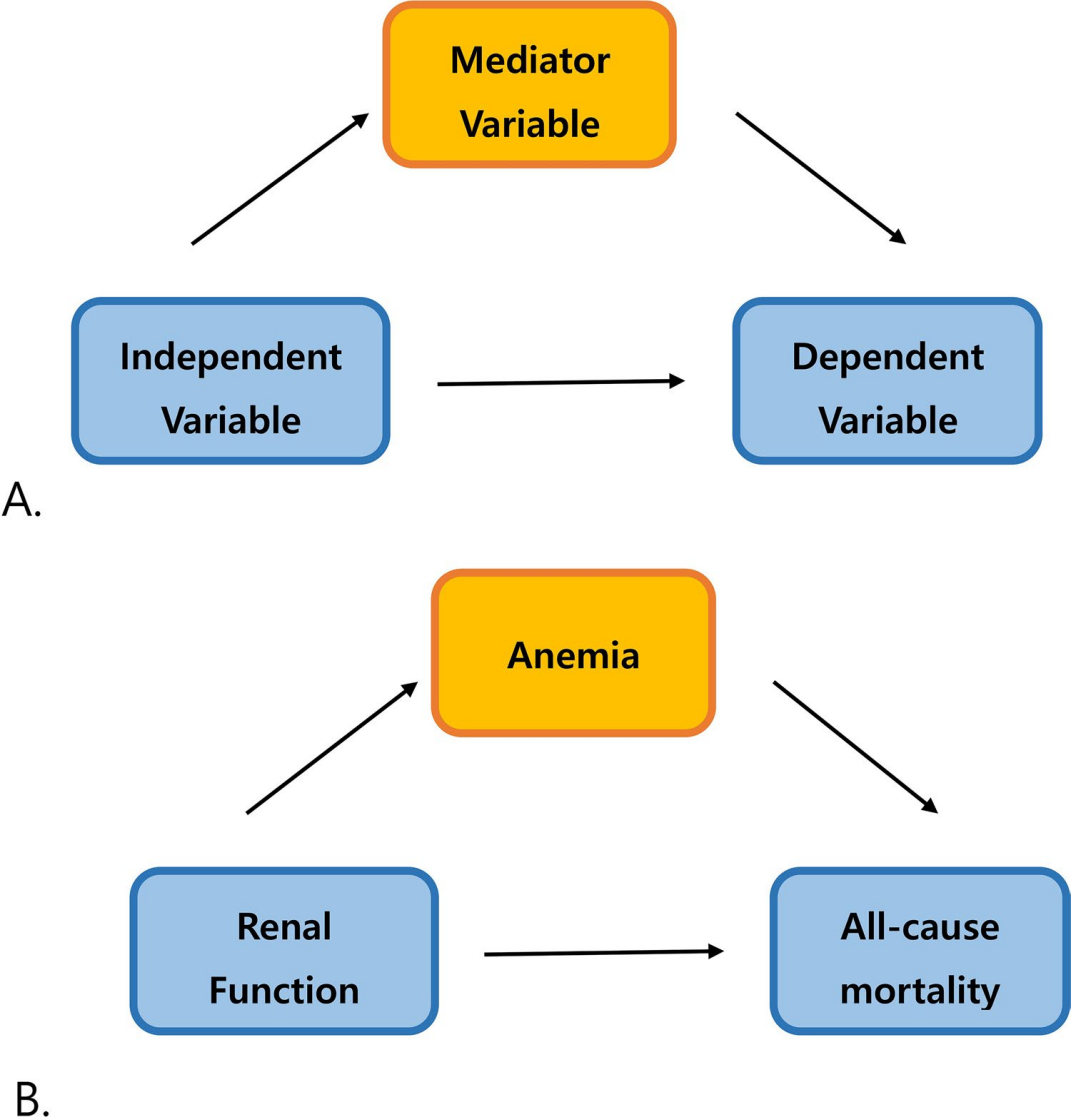
(**A**) The concept of mediation analysis. (**B**) The association between renal function and all-cause mortality mediated by anemia indicators.

Mediation analyses are usually employed to examine a causal relationship by exploring the underlying mechanism or process by which one variable influences another variable through a mediating variable^15^. In particular, mediation analysis can contribute to a better understanding of the relationship between an independent variable and a dependent variable when these variables do not have an obvious direct connection and identify possible intervention points and benefits of interventions, as shown in Fig. 1A^16^.

Based on National Health and Nutrition Examination Survey (NHANES) data, we aimed to evaluate the mediating effects of anemia indicators on kidney dysfunction and death in patients.

## Materials and methods

### Study design and population

The NHANES is a large, serial, cross-sectional survey designed to provide estimates of common chronic conditions and associated risk factors using a representative sample of the civilian, noninstitutionalized population of the United States^17,18^. We used NHANES survey data collected from 1999 through 2016. The protocol for the NHANES was approved by the National Center for Health Statistics (NCHS) Institutional Review Board^17^. For our study, we included all 44,099 participants aged 20 years or older with available estimated glomerular filtration rate (eGFR) data to allow for consistency with previous studies using the same indicator^19,20^.

The study protocol was approved by the Seoul National University Hospital and Seoul National University Boramae Medical Center ethics committee/institutional review board (IRB no. 2106-178-1230). The requirement for informed consent was waived by the Seoul National University Hospital and Seoul National University Boramae Medical Center ethics committee/institutional review board because of the retrospective nature of the study and because the analyses used anonymous and deidentified survey data. All clinical investigations were conducted in accordance with the guidelines of the 2013 Declaration of Helsinki.

### Anemia biomarkers

We assessed four blood markers that are indicative of anemia: hemoglobin [Hb; g/dl], hematocrit [Hct; %], red cell distribution width [RDW; %], and the mean corpuscular hemoglobin concentration [MCHC; g/dl]. The NHANES laboratory test data of each participant were collected. The samples were measured, processed, stored, shipped and analyzed according to a standardized protocol^21^.

### Renal function

As a biomarker that indicates renal function, we applied the eGFR. We calculated each participant’s eGFR using their serum creatinine level and an estimating equation developed by the Chronic Kidney Disease Epidemiology Collaboration (CKD-EPI) in 2021^22–24^.

### Covariates

We considered demographic variables (age, sex, and race), health behaviors (current smoking status [yes or no] and current drinking status, which was based on a questionnaire regarding the consumption of at least 12 alcoholic drinks per year [yes, no, and unknown]), comorbidities (diabetes and hypertension [yes or no, each]), body mass index (BMI), laboratory variables (hemoglobin [Hb], hematocrit [Hct], mean corpuscular volume [MCV], mean corpuscular hemoglobin [MCH], eGFR, albumin and iron), and family income-to-poverty ratio as potential confounders in the main analysis. To consider potentially nonlinear confounding effects, the age variable was adjusted as a categorical variable: 20–45 years, 46–64 years, and 65 years or older (age groups). Participants’ demographic variables, family income-to-poverty ratio, alcohol consumption, smoking status, and comorbidities (e.g., diabetes, hypertension) were assessed using self-reported information, and BMI was recorded by a trained examiner in the Mobile Examination Center.

### Subpopulations

To identify different mediating effects of anemia by subpopulation, we repeated the main analysis in (1) age-stratified populations (aged 20–45 years, 46–64 years, and 65 years or older), (2) patients with anemia, and (3) patients with CKD. Patients with anemia were defined as those with an Hb level below 13.0 g/dl for men and 12.0 g/dl for women according to the World Health Organization (WHO) guidelines^25^. Additionally, age categories were defined based on the main causes of anemia that vary depending on sex and age^26,27^: 20–45 years, 46–64 years and ≥ 65 years^28,29^. Gynecological disorders and maternal hemorrhage are important contributors to the anemia burden, especially in women of reproductive age, so we divided the participants into three groups: the premenopausal, perimenopausal, and postmenopausal groups^30,31^. The equivalent age categories were used when analyzing male participants. In addition, CKD was defined as an eGFR calculated using the Chronic Kidney Disease-Epidemiology Collaboration (CKD-EPI) equation of < 60 ml/min/1.73 m^2^ according to the KDIGO 2021 clinical practice guidelines for the evaluation and management of chronic kidney disease. CKD was defined using the eGFR category only—albuminuria categories were not analyzed.

### Clinical outcomes

The outcome of this study was all-cause mortality. We used NHANES data that were linked to National Death Index (NDI) mortality data. The NDI mortality data file included cases of mortality followed-up through December 31, 2019. In addition, we did not include NHANES data collected after 2017 because we determined that a follow-up of at least 3 years was needed to examine the association of mortality with the eGFR and anemia variables. This was because most other studies on similar topics were conducted with a follow-up period of 1–3 years or more^32–34^.

### Statistical analysis

First, to show the associations of eGFR and anemia variables with mortality, we performed survey-weighted Cox proportional hazard models considering the complex, multistage, probability sampling design of NHANES (National Center for Health Statistics)^35,36^.

Then, we identified the mediating effect of a single anemia variable by comparing hazard ratios estimated from the survey-weighted Cox proportional hazard models (which consider the complex survey characteristics of NHANES)^36^ for the association between eGFR and mortality, unadjusted and adjusted for each anemia indicator in turn. For each anemia indicator, the percentage of mediation was estimated as follows: (HR − HRc)/(HR − 1) × 100%. HR is the anemia indicator-unadjusted hazard ratio, and HRc is the hazard ratio after univariable adjustment for each anemia indicator. To adjust for potential residual confounding caused by a temporal trend, we included indicator variables for study years. The 95% confidence intervals for the estimated percentage mediation were obtained using a 500-iteration bootstrap resampling procedure.

In addition, the combined mediating effect of multiple anemia variables was calculated using the same procedures. Multiple mediator models (i.e., multivariable adjustment models) were built by selecting the anemia variable from the univariable-adjustment models. We tried all combinations of significant anemia variables and displayed the results based on the size of the mediation effect (%), shown in Fig. 1B. However, Hct was excluded across all multiple mediator models to avoid a potential nonidentifiability problem with Hb (due to the very high correlation between Hct and Hb).

Finally, we used R software (version 4.3.2) to perform all statistical analyses.

## Results

### Baseline characteristics of the study population

In our analysis, we included 44,099 individuals from the NHANES surveys conducted from 1999 through 2016. The baseline characteristics and demographic data of the study group are listed in Table 1. The mean age of the 44,099 participants was 49.44 years; the proportion of men was 48.3% (n = 21,288). The mean eGFR was 94.5 ml/min/1.73 m^2^. The patients with CKD accounted for 8.2% of the sample (n = 3614). Among the 44,099 participants, 4727 (10.7%) had anemia. The group with anemia had a higher number of older and female individuals, as well as a higher proportion of individuals with comorbidities, such as hypertension, diabetes and CKD, than the group without anemia.

**Table 1:** Baseline characteristics of the study population.

Characteristic	Total (N = 44,099)	Anemia (N = 4727)
Age (years)		
20–45	19,965 (45.3)	1912 (40.4)
46–64	13,360 (30.3)	1102 (23.3)
64 or older	10,774 (24.4)	1713 (36.2)
Sex		
Male	21,288 (48.3)	1520 (32.2)
Female	22,811 (51.7)	3207 (67.8)
Race/Hispanic origin		
Mexican American	8040 (18.2)	724 (15.3)
Other Hispanic	3580 (8.1)	360 (7.6)
Non-Hispanic White	20,152 (45.7)	1392 (29.4)
Non-Hispanic Black	8773 (19.9)	1868 (39.5)
Other race	3554 (8.1)	383 (8.1)
BMI (kg/m^2^)		
< 18.5	686 (1.6)	73 (1.5)
18.5–25	12,310 (27.9)	1291 (27.3)
25–30	14,811 (33.6)	1350 (28.6)
30 or over	15,529 (35.2)	1840 (38.9)
Comorbidities		
Diabetes	6247 (14.2)	1135 (24.0)
Hypertension	18,313 (41.5)	2441 (51.6)
Current smoking		
No	23,790 (53.9)	2909 (61.5)
Yes	20,309 (46.1)	1818 (38.5)
Current drinking*		
Yes	28,471 (64.6)	2468 (52.2)
No	12,081 (27.4)	1769 (37.4)
Unknown	30 (0.1)	4 (0.1)
Laboratory tests		
Hemoglobin (Hb; g/dl)	14.1 (1.6)	11.4 (1.0)
Hematocrit (Hct;%)	41.6 (4.4)	34.4 (2.9)
Mean corpuscular hemoglobin (MCV; fL)	89.5 (5.8)	85.2 (9.4)
Mean corpuscular hemoglobin (MCHC; pg)	30.4 (2.4)	28.4 (3.8)
Albumin (g/dl)	4.2 (0.4)	3.9 (0.4)
eGFR (ml/min/1.73 m^2^)	94.5 (23.2)	87.0 (32.1)
Family income to poverty ratio	2.5 (1.6)	2.2 (1.5)
Patients with anemia	4727 (10.7)	–
Patient with early CKD	15,645 (35.5)	4727 (21.2)
Patients with CKD	3614 (8.2)	1048 (22.2)

The data are presented as the mean ± standard deviation or number (%). *Current drinking status was based on a questionnaire regarding the consumption of at least 12 alcoholic drinks per year.

*BMI* body mass index, *Egfr* estimated glomerular filtration rate.

Early CKD patients were grouped according to the following criteria: eGFR ≥ 90 ml/min/1.73 m^2^ and ACR ≥ 30 or 60 ≤ eGFR ≤ 90. CKD was defined as an eGFR ≤ 60 ml/min/1.73 m^2^ in this study. Anemia was defined as a Hb level less than 13.0 g/dl for men and 12.0 g/dl for women according to the World Health Organization (WHO) guidelines.

### Association between eGFR and mortality

**Figure 2:**
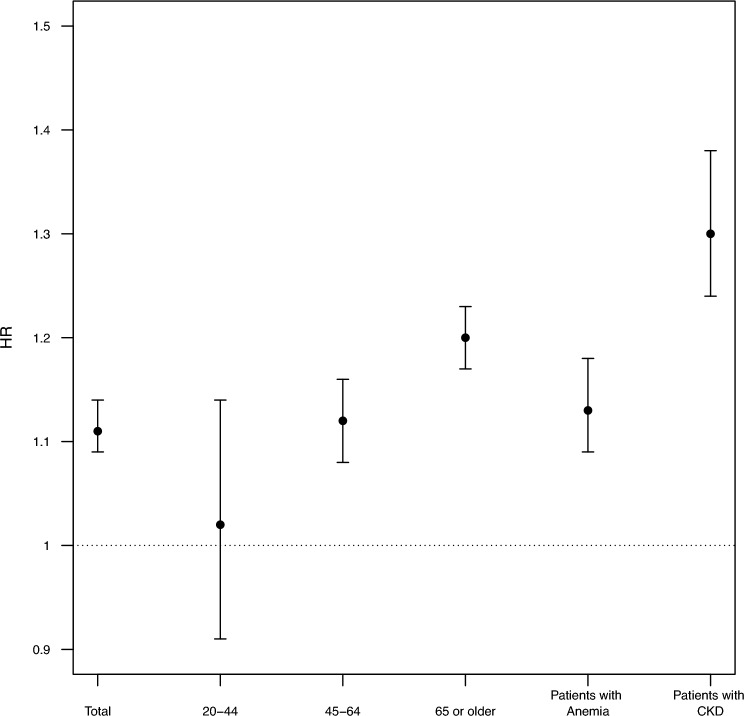
The association between eGFR and mortality. *eGFR* estimated glomerular filtration rate. The associations are displayed as hazard ratios(HR) per 1 unit increase in an anemia biomarker. Values: HRs for a 10 ml/min/1.73 m^2^ decrease in eGFR.

The association between eGFR and all-cause mortality is shown in Fig. 2 and Supplemental Table 1. The HRs for all-cause mortality per 10 ml/min/1.73 m^2^ decrease in eGFR was 1.11 (95% CI 1.09–1.13) in the total population and 1.02 (95% CI 0.91–1.14), 1.12 (95% CI 1.108–1.16) and 1.20 (95% CI 1.17–1.23) in people aged 20–45, 46–64, and ≥ 65 years, respectively. The association was more prominent in patients with anemia and patients with CKD than in the total population: the estimated HRs per 10 ml/min/1.73 m^2^ decrease in eGFR were 1.13 (95% CI 1.09–1.18) and 1.30 (95% CI 1.24–1.38) in anemia and CKD patients, respectively.

### Association between anemia indicators and all-cause mortality

The associations between anemia indicators (Hb, Hct, RDW and MCHC) and all-cause mortality were analyzed (Table 2); all the anemia indicators, except for RDW, were negatively associated with all-cause mortality in the total population: HRs (hazard ratios) 0.93 (95% CI 0.90–0.96) for Hb, 0.98 (95% CI 0.97–0.99) for Hct, and 0.86 (95% CI 0.78–0.95) for MCHC. On the other hand, RDW showed a positive association with all-cause mortality, with HRs of 1.21 (95% CI 1.16–1.26), 1.14 (95% CI 1.09–1.20), and 1.15 (95% CI 1.11–1.19) in the ≥ 65 years, CKD, and anemia groups, respectively. Among all anemia indicators, the association with mortality was generally more pronounced in elderly individuals (people aged 65 or older) and patients with anemia or CKD.

**Table 2:** Associations between anemia biomarkers and all-cause mortality.

	Hb		Hct		RDW		MCHC	
	HR	95% CI	HR	95% CI	HR	95% CI	HR	95% CI
Total	0.93	**0.90–0.96**	0.98	**0.97–0.99**	1.23	**1.20–1.27**	0.86	**0.78–0.95**
Age groups								
20–45	1.01	0.83–1.24	1.01	0.94–1.08	1.34	**1.23–1.47**	0.81	0.58–1.12
46–64	0.95	0.89–1.01	0.98	0.96–1.00	1.26	**1.22–1.31**	0.55	0.30–1.00
65 or older	0.87	**0.84–0.90**	0.95	**0.94–0.97**	1.21	**1.16–1.26**	0.93	0.74–1.16
eGFR groups								
Early CKD	0.92	**0.88–0.96**	0.97	**0.96–0.99**	1.24	**1.19–1.29**	0.86	**0.76–0.97**
CKD	0.92	**0.88–0.97**	0.97	**0.96–0.99**	1.14	**1.09–1.20**	1.24	0.67–2.31
Hb groups								
Anemia	0.79	**0.72–0.87**	0.93	**0.90–0.96**	1.15	**1.11–1.19**	0.75	0.42–1.32
No anemia	1.01	0.97–1.05	1.00	0.99–1.02	1.28	**1.23–1.32**	0.92	0.78–1.08

The associations are displayed as hazard ratios(HR) per 1 unit increase in an anemia biomarker.

Definitions: HRs for a 1-unit increase in each mediator. Early CKD patients were grouped according to the following criteria: eGFR ≥ 90 ml/min/1.73 m^2^ and ACR ≥ 30 or 60 ≤ eGFR ≤ 90. CKD was defined as an eGFR ≤ 60 ml/min/1.73 m^2^ in this study. Anemia was defined as a Hb level less than 13.0 g/dl for men and 12.0 g/dl for women according to the World Health Organization (WHO) guidelines.

The bold text indicates an evident effect according to the 95% confidence interval.

*HR* hazard ratio, *CI* confidence interval, *CKD* chronic kidney disease, *Hb* hemoglobin, *Hct* hematocrit, *RDW* red cell distribution width, *MCHC* mean corpuscular hemoglobin concentration, *eGFR* estimated glomerular filtration rate, *ACR* albumin–creatinine ratio.

### Mediating effects in patient subgroups

In the total population, three anemia biomarkers (Hb, Hct, and RDW) individually mediated the association between eGFR and all-cause mortality (Table 3), and the estimated univariable mediating effects of Hb, Hct, and RDW were 14.48 (95% CI 8.62–21.07%), 15.14 (9.22–21.07%) and 36.48 (29.96–43.01%), respectively. The proportion of mediation by anemia variables was generally more evident in elderly (people aged ≥ 65 years) and participants with CKD or anemia than in the total population: in patients aged ≥ 65 years, the univariable mediating effects of Hb, Hct and RDW were 22.70% (18.09–27.32%), 22.71% (18.33–27.09%) and 25.11% (20.56–29.66%), respectively; in patients with CKD, the mediating effects were 17.55 (16.52–18.58%), 17.60 (16.36–18.84%), and 36.19 (34.76–37.61%), respectively; and in patients with anemia, the mediating effects were 29.30 (27.98–30.62%), 28.28 (27.19–30.62%), and 13.04 (12.01–14.06%), respectively.

**Table 3:** Univariate mediation analysis revealed the mediating role of anemia biomarkers in the association between the eGFR and all-cause mortality.

	Hb		Hct		RDW		MCHC	
	Mediation (%)	95% CI	Mediation (%)	95% CI	Mediation (%)	95% CI	Mediation (%)	95% CI
Total	14.84	**8.62 to 21.07**	15.14	**9.22 to 21.07**	36.48	**29.96 to 43.01**	0.21	− 6.12 to 6.53
Age groups (years)								
20–45	152.48	NA*	26.26	NA	88.42	NA	− 56.37	NA
46–64	8.15	− 1.30 to 17.60	2.62	− 2.01 to 9.21	16.3	**4.62 to 36.12**	− 1.67	6.55 to 1.34
65 or older	22.70	**18.09 to 27.32**	22.71	**18.33 to 27.09**	25.11	**20.56 to 29.66**	0.14	− 4.47 to 4.75
eGFR groups								
Early CKD	12.20	**10.90 to 13.50**	11.88	**10.70 to 13.07**	− 3.32	− 4.69 to − 1.95	− 0.23	− 1.48 to 1.01
CKD	17.55	**16.52 to 18.58**	17.60	**16.36 to 18.84**	36.19	**34.76 to 37.61**	0.15	− 1.01 to 1.31
Hb groups								
Anemia	29.30	**27.98 to 30.62**	28.28	**27.19 to 30.62**	13.04	**12.01 to 14.06**	− 1.06	− 1.96 to − 0.16
No anemia	-0.22	− 0.52 to 0.07	− 0.19	− 0.58 to 0.20	43.11	**42.72 to 43.49**	− 0.04	− 0.38 to 0.30

The mediation % presents the proportion of all mediating effects attributed to an anemia biomarker, for the association between CKD and all-cause mortality.

The bold text indicates a statistical significance according to the 95% confidence interval.

*CKD* chronic kidney disease, *Hb* hemoglobin, *Hct* hematocrit, *RDW* red cell distribution width, *MCHC* mean corpuscular hemoglobin concentration, *eGFR* estimated glomerular filtration rate.

*NA: not applicable due to the convergence problem (the confidence interval could not be estimated from the resampling method due to the insufficient sample size and the resultant convergence problem in the Cox proportional hazard model).

**Figure 3:**
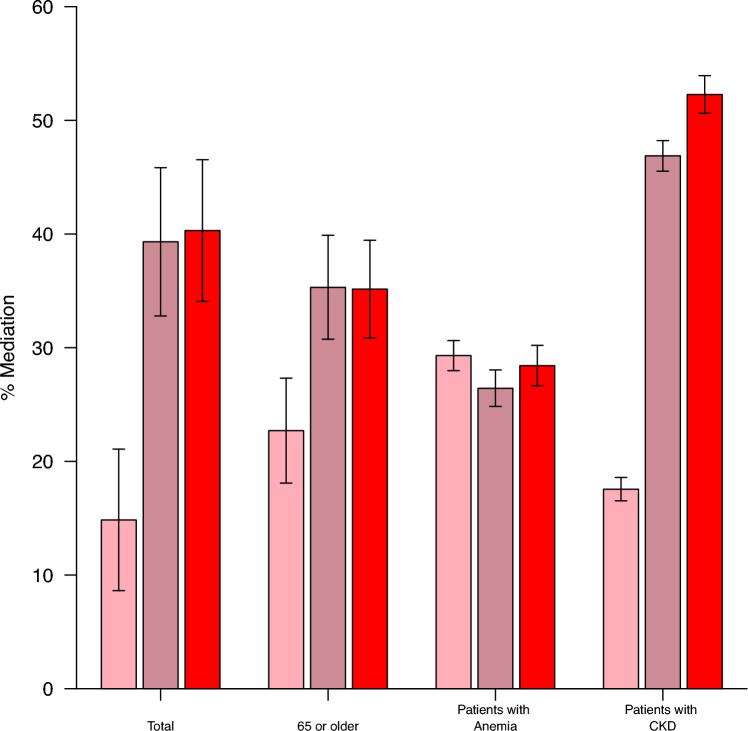
Percentage of the mediating effect of potential mediators on all-cause mortality across patient subgroups. The mediation % presents the proportion of all mediating effects attributed to an anemia biomarker, for the association between CKD and all-cause mortality. Definitions: HRs for a 1-unit increase in each mediator. Early CKD was grouped as eGFR ≥ 90 ml/min/1.73 m^2^ and ACR ≥ 30 or 60 ≤ eGFR ≤ 90. CKD was defined as eGFR ≤ 60 ml/min/1.73 m^2^ in this study. Anemia was defined as an Hb level below 13.0 g/dl for men and 12.0 g/dl for women according to the WHO (World Health Organization) guidelines.

The combined mediating effect of the three anemia indicators (Hb, RDW, and MCHC) on the association between kidney function and all-cause mortality was estimated as 40.30% (95% CI 34.07–46.54%) in the total population (Fig. 3; Supplemental Table 2). Moreover, the combined mediating effect was statistically significant in the subgroups of individuals aged > 65 years (35.15% with 95% CI 30.85–39.45%), patients with anemia (28.42% with 95% CI 26.64–30.21), and patients with CKD (52.27% with 95% CI 50.61–53.93) than in the total population. This finding shows that the CKD population was more susceptible to anemia than the general population, although anemia was one of the significant mediators in both groups.

## Discussion

In this study, we investigated the potential mediating roles of anemia indicators (Hb, Hct, RDW, and MCHC) in the association between kidney function and mortality using nationally representative NHANES data including 44,099 adults between 1999 and 2016. We found that anemia indicators might mediate the association between kidney function and all-cause mortality in the total population, and the proportion mediated by anemia indicators was generally greater in the elderly population and patients with anemia or CKD.

Many previous studies have reported the relationship between anemia and CKD prognosis^37–39^ and the association between kidney function and mortality^40,41^; however, it has been unclear whether the association is mediated by anemia variables and what proportion of the association can be explained by the mediation. Our analyses improve the understanding of the potential mediating roles of anemia indicators on the relationship between kidney function and mortality and provide epidemiological evidence of relevant possible intervention strategies^16^. Moreover, the disparity in magnitude of the proportion mediated by anemia-related variables across subgroups (higher mediating % in elderly individuals and participants with anemia or CKD) suggests that target-specific anemia interventions may be more effective if they are developed with consideration of individual-level characteristics.

Interestingly, compared to other anemia indicators, RDW had a greater impact on mortality. In addition, although the clinical usefulness of RDW in kidney disease has been limited, our findings suggest the relevance of RDW as a promising parameter in CKD patients and quantified the effect of anemia indicators on CKD patients through mediation analysis. The anemia indicators (Hb, Hct, RDW, MCHC) assessed in our study are inexpensive and easily obtainable parameters that can predict the probability of adverse outcomes in patients with CKD. Hence, in CKD patients with anemia, anemia indicators (Hb, Hct, RDW, MCHC) should be more closely monitored and managed to improve all-cause mortality.

The RDW represents the number of circulating erythrocytes^42^. Clinically, the RDW is helpful as a prognostic indicator of acute diseases such as sepsis and pancreatitis^43,44^. Lippi et al.^45^ showed a strong association between RDW and eGFR values in a cross-sectional study. Patients with higher RDW values have impaired renal function. Nabais et al.^46^ also showed that a higher RDW value was associated with both 6-month overall mortality and 6-month mortality related to acute coronary syndrome (ACS). Recently, we also showed that an increased time-averaged RDW value was significantly associated with increased mortality in CKD patients aged > 45 years^47^. The results of our study were similar to those of previous studies on the reverse relationships between the RDW and mortality.

A possible pathophysiological mechanism is that the RDW value is associated with endothelial dysfunction and inflammation, which increases the occurrence of severe morbidity, including deteriorated renal function and death^48^. Some studies also showed a significant positive correlation between the RDW and serum C-reactive protein (CRP) level, which indicates inflammation^45,46^. Although the RDW could provide useful additional information in the clinic, its role is still controversial because it is a nonspecific diagnostic tool^49^.

Although there are several classical pharmaceutical interventions to improve CKD anemia, such as erythropoiesis-stimulating agents and/or iron supplementation^50^, there are a lack of studies focused on reducing the RDW value to prevent disease progression or even reduce all-cause mortality. Recent studies on potential anemia treatment have targeted the inhibition of hepcidin production to improve inflammation status^51^. Such trials have resulted in lower levels of RDW. Hence, our study indicated that further studies investigating the potential therapeutic effect of reducing RDW in CKD patients with anemia would be valuable.

In addition, we explored the association between eGFR and all-cause mortality. We demonstrated that lower eGFR predicted worse prognosis in all age groups except for the young age group (20–45 years group). In addition, the CKD group, identified as those with an eGFR lower than 60 ml/min/1.73 m^2^, showed a strong positive association with mortality compared to the early CKD group, which was characterized by an eGFR lower than 90 ml/min/1.73 m^2^ or an eGFR greater than 90 ml/min/1.73 m^2^ with microalbuminuria. Our finding of the relationship of increasing age in CKD patients above 45 years with higher mortality is similar to that of previous studies^52,53^. In a USRDS study, each 1-year increase in age was associated with an independent risk factor for 3-month mortality^53^. A possible reason for this finding is the presence of many comorbidities in later stages of CKD and old age, although this is insufficient to explain the factors that were associated with different CKD stages and progression of CKD in this study.

However, our study has several limitations. This was an observational cohort study, not a double-blind randomized controlled study. Thus, the results of this study should be interpreted as associations rather than not causal relationships. Second, there was a lack of analysis of outcomes other than all-cause mortality due to limited access to data. Third, we investigated cross-sectional data, so it was difficult to follow up on outcomes other than death, such as serial changes in eGFR and nonfatal cardiovascular disease. Long-term follow-up data are necessary to clarify the mediating effects of anemia. Fourth, we estimated the statistically significant effect on the eGFR—mortality relationship in the early CKD group by removing the mediating effect of the RDW (Table 3). This result should be interpreted with caution because there was nearly a negative and not a statistically significant association between the eGFR and mortality in the early CKD group. In this case, the estimated mediating effect (%) is less meaningful, and the interpretation of the mediating effect (%) was not epidemiologically suitable because the denominator of the mediating effect (%) (the eGFR—mortality relationship) was estimated to be zero. In this group, because the association between the RDW and mortality was statistically significant and positive (Table 2), this result should be interpreted as showing only a RDW—mortality association, not as the mediating role. Finally, the use of erythrocyte-stimulating agents is also a limitation that may affect the RDW values, but most of the patients with advanced CKD receive these medications during anemic intervals. Thus, the results should be interpreted with caution. Nevertheless, the strength of this study is that we analyzed data from a large national survey that represent the population, so the study may be less prone to institutional and selection biases. Furthermore, we used a new analytic technique, mediation analysis, that is a potential method to quantify the influence of potential mediators and presented a new direction for statistical analysis.

In conclusion, we quantified the mediating effects of anemia on all-cause mortality in CKD patients by conducting mediation analysis, a new statistical analysis method. Additionally, our study proposes possible mechanisms by which anemia indicators might affect CKD prognosis.

### Supplementary Information

Supplementary Information.

## Data Availability

All data analyzed during the current study are available online on the National Center for Health Statistics website (https://www.cdc.gov/nchs/nhanes).
