# Supplementary material for: The estimated mediating roles of anemia-related variables in the association between kidney function and mortality: a National Health and Nutrition Examination Survey (NHANES) study
Source: Sci Rep. 2024 Mar 19;14:6621. doi: 10.1038/s41598-024-56877-7 (PMC10951385; doi:10.1038/s41598-024-56877-7)
Supplement: Supplementary file 1 — Supplementary Information. [file 41598_2024_56877_MOESM1_ESM.docx]

**Supplementary Materials and Table of Contents**

**Supplemental Table 1.** Association between the eGFR and mortality

**Supplemental Table 2.** Multivariate analysis of the associations between anemia biomarkers and all-cause mortality

**Supplementary Table 1.** Association between the eGFR and mortality

|  | **eGFR (per 10-unit decrease)** | |
| --- | --- | --- |
|  | HR | 95% CI |
| **Total** | 1.11 | **1.09-1.14** |
| **Age groups (years)** |  |  |
| 20-45 | 1.02 | 0.91-1.14 |
| 46-64 | 1.12 | **1.08-1.16** |
| 65 or older | 1.18 | **1.15-1.20** |
| **eGFR groups** |  |  |
| Early CKD | 0.95 | 0.90-1.00 |
| CKD | 1.30 | **1.24-1.38** |
| **Hb groups** |  |  |
| Anemia | 1.11 | **1.07-1.15** |
| No anemia | 1.09 | **1.07-1.12** |

Abbreviations: HR, hazard ratio; CI, confidence interval; CKD, chronic kidney disease; Hb, hemoglobin; Hct, hematocrit; RDW, red cell distribution width; MCHC, mean corpuscular hemoglobin concentration; eGFR, estimated glomerular filtration rate; ACR, albumin–creatinine ratio.

Definitions: HRs for a 1-unit increase in each mediator. Early CKD patients were grouped according to the following criteria: eGFR ≥90 mL/min/1.73 m^2^ and ACR ≥30 or 60 ≤ eGFR ≤ 90. CKD was defined as an eGFR ≤60 mL/min/1.73 m^2^ in this study. Anemia was defined as a Hb level less than 13.0 g/dl for men and 12.0 g/dl for women according to the World Health Organization (WHO) guidelines.

The bold text indicates an evident effect according to the 95% confidence interval.

**Supplementary Table 2.** Multivariate mediation analysis revealed the mediating role of anemia biomarkers in the association between the eGFR and all-cause mortality.

|  | **Model 1** | | **Model 2** | |
| --- | --- | --- | --- | --- |
|  | **% Mediation** | **95% CI** | **% Mediation** | **95% CI** |
| Total | 39.67 | 33.24-46.09 | 40.30 | 34.07-46.54 |
| 65 years or older | 35.31 | 30.74-39.86 | 35.15 | 30.85-39.45 |
| Anemia | 26.43 | 24.83-28.04 | 28.42 | 26.64-30.21 |
| CKD | 46.87 | 45.52-48.21 | 52.27 | 50.61-53.93 |

Model 1: Adjusted for the hemoglobin level and red cell distribution width (RDW)

Model 2: Adjusted for the Hb level, RDW and mean corpuscular Hb concentration (MCHC)

Abbreviations: CI, confidence interval; CKD, chronic kidney disease.

Definitions: CKD was defined as an eGFR ≤60 mL/min/1.73 m2 in this study.
